# Challenges in causality assessment of renal dysfunction during sequential treatment for presumed rifampicin-resistant tuberculosis in an elderly patient

**DOI:** 10.1016/j.idcr.2026.e02758

**Published:** 2026-09-09

**Authors:** Thi Thanh Thuy Hoang, Hoang Chinh Ha, Han Thi Nguyen, Thien Huong Nguyen, Andrew James Codlin, Luan Nguyen Quang Vo

**Affiliations:** a National TB Control Programme of Vietnam, National Lung Hospital, Hanoi, Viet Nam; b Soc Trang Lung Hospital, Viet Nam; c Research Department, FIT RD Social Enterprise Company Limited (FIT RD), Hanoi, Viet Nam; d Division of Infectious Diseases, Department of Medicine Solna, Karolinska Institutet, Stockholm, Sweden; e KNCV Tuberculosis Foundation, Viet Nam; f Friends for International TB Relief, Hanoi, Viet Nam; g Department of Global Public Health, Karolinska Institutet, Stockholm, Sweden

**Keywords:** Acute kidney injury, Rifampicin-resistant tuberculosis (RR-TB), Causality assessment, Drug rechallenge, BPaLM

## Abstract

**Background:**

Renal impairment during treatment for rifampicin-resistant tuberculosis (RR-TB) is uncommon but clinically significant, especially in elderly patients with limited physiological reserve. Determining whether renal dysfunction is attributable to anti-tuberculosis drugs or patient-related factors remains a major clinical challenge, particularly following sequential regimen changes.

**Case presentation:**

A 74-year-old man with a low body mass index (17.5 kg/m²) was treated for presumed RR-TB based on a single Xpert MTB/RIF assay showing rifampicin resistance despite persistently negative smear microscopy and mycobacterial culture. Baseline renal function was normal (serum creatinine 89 µmol/L; estimated glomerular filtration rate [eGFR] 73.2 mL/min/1.73 m²). Eighteen days after initiation of a long oral regimen (D2: levofloxacin, clofazimine, linezolid, pyrazinamide, and cycloserine), acute kidney injury developed, with serum creatinine increasing to 140 µmol/L and eGFR declining to 42.34 mL/min/1.73 m². The regimen was switched to BPaLM, during which renal function worsened with marked fluctuations (eGFR 24.65–57.3 mL/min/1.73 m²)**,** requiring supportive care**.** Renal function partially improved after treatment interruption. Rechallenge with the D2 regimen was associated with relatively less fluctuation in renal function. WHO–UMC causality assessment classified the relationship between anti-tuberculosis treatment and renal dysfunction as "possible."

**Conclusion:**

This case highlights the complexity of evaluating renal dysfunction during sequential treatment for RR-TB. Advanced age, low BMI, frailty, and fluctuating hydration status should be considered when interpreting changes in renal function. Careful interpretation requires integration of the clinical course and available diagnostic information rather than reliance on temporal associations alone.

## Introduction

Rifampicin-resistant tuberculosis (RR-TB) often requires prolonged treatment with second-line anti-tuberculosis drugs and is associated with more frequent adverse events than drug-susceptible tuberculosis treatment [1]. Although nephrotoxicity is less common than hematologic, hepatic, or neurologic toxicity, renal impairment remains clinically important, particularly in elderly patients and those with limited physiological reserve [2]. Beyond its potential impact on treatment continuity, renal dysfunction may complicate clinical decision-making because it is often difficult to determine whether deterioration in kidney function is attributable to anti-tuberculosis drugs, underlying patient characteristics, or intercurrent clinical conditions [3].

Recent World Health Organization guidelines recommend all-oral regimens, including BPaLM (bedaquiline, pretomanid, linezolid, and moxifloxacin), as preferred treatment options for RR-TB [4]. Despite reducing exposure to injectable agents, renal dysfunction may still occur during treatment with all-oral regimens, and its underlying cause is often difficult to determine, particularly when multiple potential contributing factors coexist. However, real-world data regarding renal safety during treatment with these regimens remain limited. Distinguishing drug-induced kidney injury from multifactorial renal dysfunction is challenging because of nonspecific manifestations, heterogeneous mechanisms, and multiple predisposing factors, especially in older adults with reduced renal reserve [3]. This challenge may become even greater when patients require sequential regimen modifications because changes in renal function cannot always be readily attributed to the newly introduced regimen alone.

Assessment of adverse drug reaction causality is commonly performed using the WHO–Uppsala Monitoring Centre (WHO–UMC) system and the Naranjo algorithm [5], [6]. The Naranjo algorithm applies a structured scoring method based on factors such as temporal association, rechallenge, and alternative causes [5], but may be limited in complex clinical settings with incomplete data or multiple concomitant drugs [7]. In contrast, the WHO–UMC system allows greater flexibility and broader clinical judgment in pharmacovigilance assessments [6], [7]. Although these tools provide a structured framework, their interpretation should be integrated with the overall clinical context, particularly when multiple potential contributing factors coexist.

Here, we report a case of fluctuating renal dysfunction during treatment for presumed RR-TB in an elderly patient receiving both a long oral regimen (D2) and a subsequent BPaLM regimen. The causal relationship between anti-tuberculosis therapy and renal dysfunction was classified as “possible” using the WHO–UMC system. This case highlights the complexity of causality assessment for renal dysfunction during sequential treatment for RR-TB and the importance of individualized clinical evaluation, monitoring, and supportive management.

## Case presentation

A 74-year-old man with a history of bacteriologically confirmed pulmonary tuberculosis without rifampicin resistance detected by Xpert MTB/RIF assay had been successfully treated with first-line anti-tuberculosis treatment (SHRZE) in April 2018. In November 2024, he was admitted for management of chronic obstructive pulmonary disease and residual post-tuberculosis fibrotic lung disease.

The patient presented with mild productive cough and reduced appetite. Physical examination revealed bilateral wheezing and crackles. His body weight was 45 kg and his height was 160 cm (BMI 17.5 kg/m²). Sputum smear microscopy was negative, and no additional mycobacterial tests were performed during that hospitalization.

**Fig. 1:**
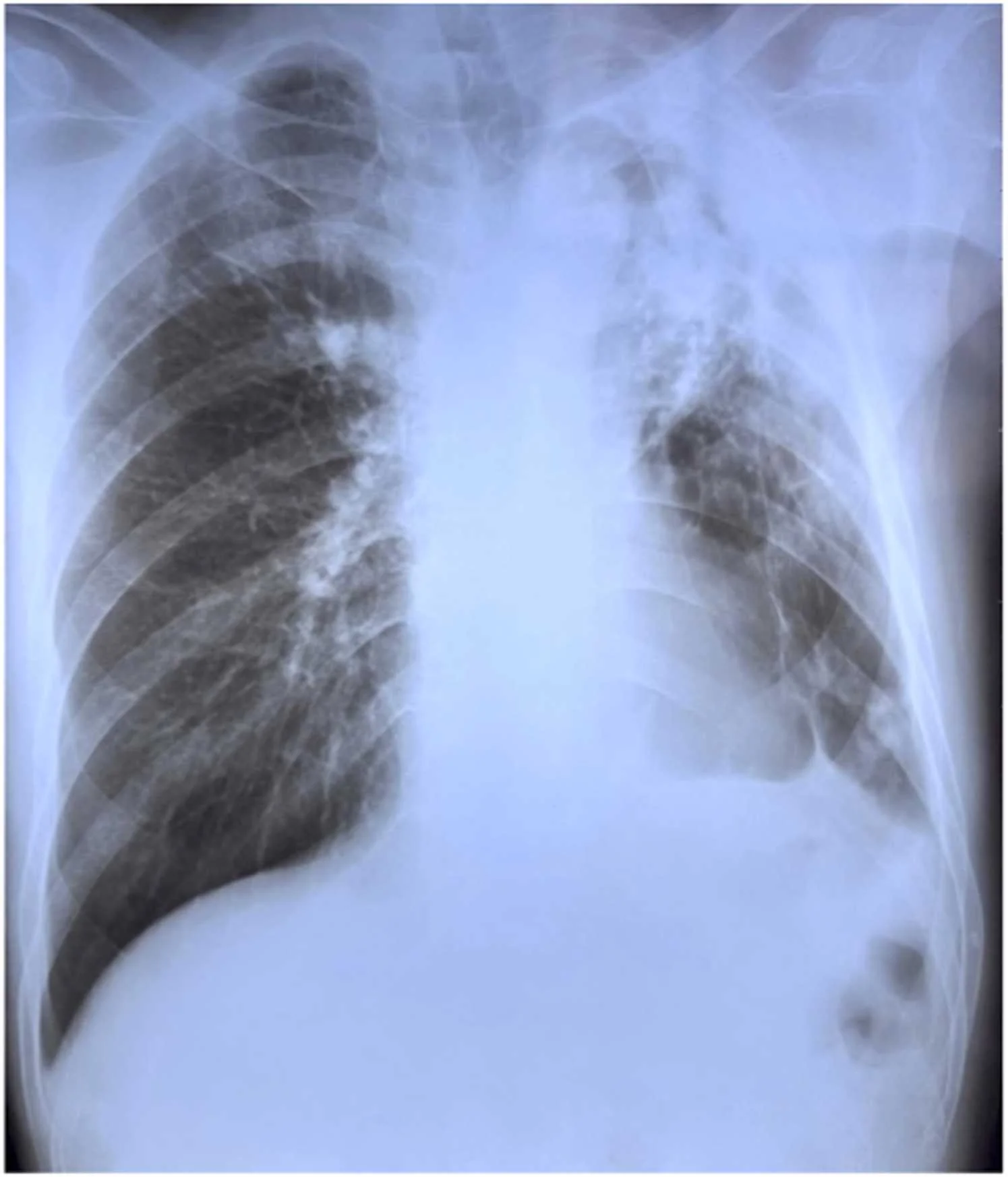
Chest X-ray before treatment.

In January 2025, a single Xpert MTB/RIF assay reported detection of *Mycobacterium tuberculosis* with a very low bacterial load and rifampicin resistance. Baseline acid-fast bacilli smear microscopy and mycobacterial culture were negative and remained negative during follow-up. Chest radiography showed chronic post-tuberculosis structural abnormalities, including fibrotic infiltrates, volume loss, tracheal deviation, and elevation of the left hemidiaphragm, while active tuberculosis superimposed on these chronic abnormalities could not be excluded (Fig. 1). In the clinical context, the patient was managed as presumed RR-TB despite limited microbiological confirmation.

Baseline renal function on January 10, 2025, showed a serum creatinine level of 89 µmol/L and an estimated glomerular filtration rate (eGFR) of 73.2 mL/min/1.73 m².

### Initial treatment with the D2 regimen

Because BPaLM drugs were unavailable at treatment initiation, the patient received a long oral regimen (D2) consisting of levofloxacin, clofazimine, linezolid, pyrazinamide, and cycloserine. Despite initially normal renal function, by treatment day 18, serum creatinine increased to 140 µmol/L, with eGFR declining to 42.34 mL/min/1.73 m², fulfilling diagnostic criteria for acute kidney injury (AKI). Given the temporal relationship between D2 initiation and renal dysfunction, and because BPaLM drugs had become available, treatment was switched to the BPaLM regimen.

### Treatment with the BPaLM regimen

Following the switch on treatment day 22, intravenous fluid therapy was administered with gradual renal improvement. Given the need to maintain an effective RR-TB regimen, BPaLM was continued with close monitoring of renal function and supportive management. By day 30, serum creatinine decreased to 114 µmol/L and eGFR improved to 54.28 mL/min/1.73 m². However, renal function remained unstable during days 31–98, with eGFR fluctuating between 24.65 and 57.3 mL/min/1.73 m², requiring three hospital admissions.

The most severe episode was classified as grade 4 renal impairment, with eGFR declining to 24.65 mL/min/1.73 m². This renal SAE was reported to the national pharmacovigilance system through the active drug safety monitoring (aDSM). Anti-tuberculosis treatment was temporarily discontinued and supportive therapy, including intravenous fluids, was provided. Over the following two weeks (days 99–113), eGFR gradually improved to 51.5–66.8 mL/min/1.73 m².

### Reintroduction of the D2 regimen

**Fig. 2:**
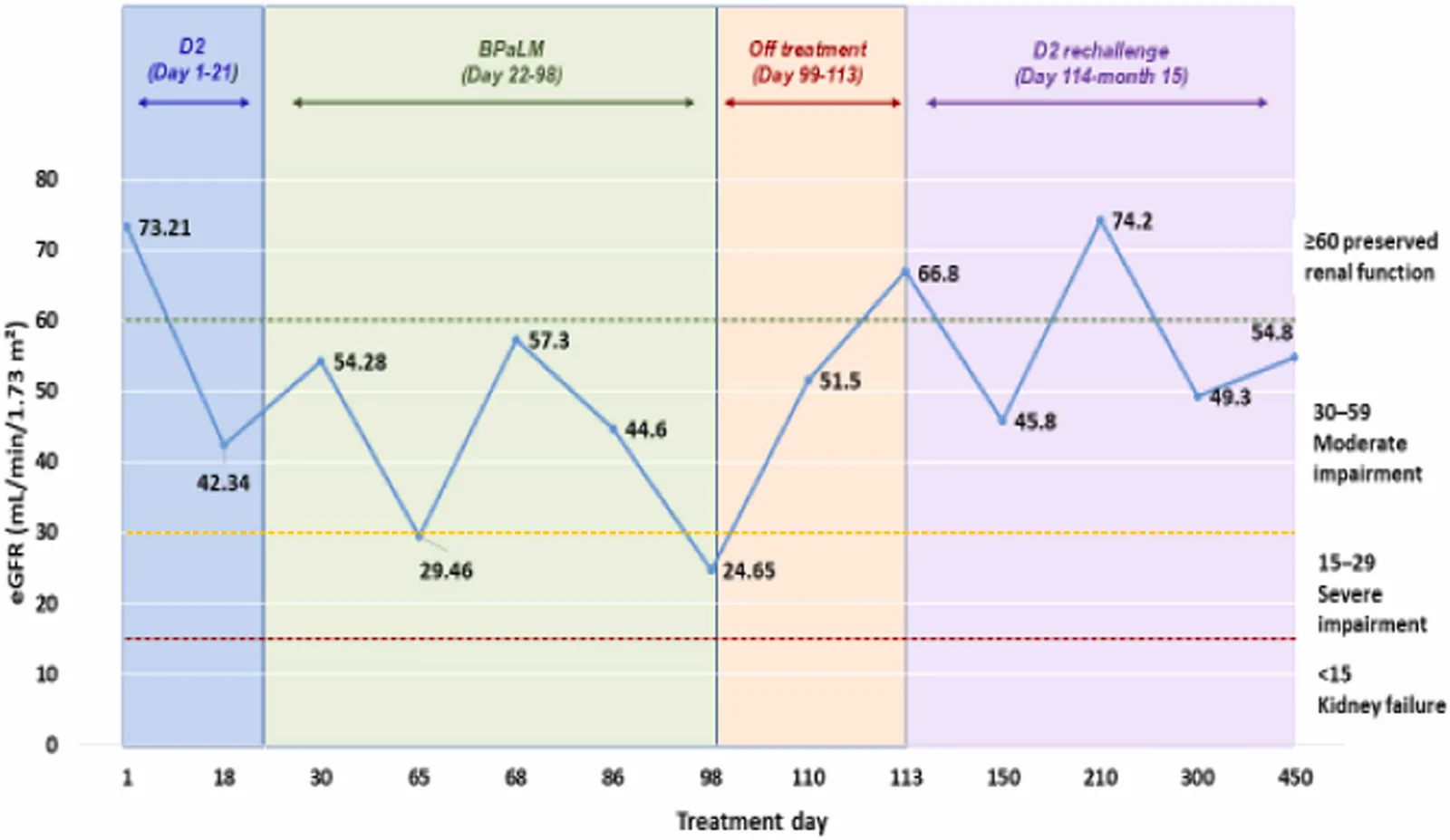
Estimated glomerular filtration rate (eGFR) over time during D2 and BPaLM treatment.

After partial improvement in renal function following treatment interruption and supportive management, the patient was restarted on the D2 regimen with close monitoring. This decision was made because renal dysfunction appeared to worsen during BPaLM treatment while continued treatment for presumed RR-TB was required. Serial monitoring demonstrated relatively less variability in renal function, with eGFR ranging from 45.8 to 74.2 mL/min/1.73 m² and fewer hospital admissions compared with the BPaLM period (Table 1). Nevertheless, sustained normalization of renal function was not achieved throughout treatment (Fig. 2). A chronological summary of anti-tuberculosis drug exposure, dosing, renal function, and treatment modifications is provided in Supplementary Table S1.

**Table 1:** Temporal changes in renal function parameters and clinical interventions during treatment.

Period	Regimen	Renal function (Creatinine, µmol/L; eGFR, mL/min/1.73 m²)	Clinical remarks
Baseline (Jan 2025)	D2	Cr 89; eGFR 73.2	Baseline normal
Day 18	D2	Cr 140; eGFR 42.34	Early renal impairment
Days 19–21	D2 + IV fluids	NA	Supportive therapy
Day 22	Switch to BPaLM	NA	Regimen change initiated
Day 23–98	BPaLM + IV fluids	Cr 109–219; eGFR 24.6–57.3	Transient early improvement followed by ongoing fluctuations and deterioration
Days 99–113	Drug interruption + IV fluids	Cr 96–119; eGFR 51.5–66.8	Improved renal function after treatment interruption
Day 114–Month 15	Reintroduction of D2	Cr 96–124; eGFR 45.8–74.2	Less variable renal function and fewer hospitalizations

**Abbreviations:** Cr = serum creatinine; eGFR = estimated glomerular filtration rate; IV fluids = intravenous fluids; NA = Not available.

The figure demonstrates temporal changes in eGFR across different treatment periods. Comparatively less variability in eGFR was observed during periods of treatment with the D2 regimen, whereas greater fluctuations and lower eGFR values were observed during the BPaLM treatment period.

### Causality assessment

Causality assessment of renal adverse events associated with anti-tuberculosis therapy was performed using the World Health Organization–Uppsala Monitoring Centre (WHO–UMC) system. The relationship was classified as “possible” based on the temporal association between initiation of the D2-containing regimen and renal dysfunction, together with partial improvement after discontinuation of both the D2 and BPaLM regimens with supportive management. However, interpretation was complicated by sequential regimen changes, fluctuating renal function, and confounding factors including advanced age, low BMI (17.5 kg/m²), and frailty. Rechallenge with the D2 regimen was inconclusive because renal function fluctuated without a clear trend toward further deterioration upon re-exposure. Importantly, causality assessment was applied to the overall treatment regimens rather than to any single individual drug.

During expert review, levofloxacin and pyrazinamide in the D2 regimen were considered the most likely suspected agents based on their reported association with renal adverse events. However, the contribution of individual drugs could not be established. The BPaLM components were considered less likely contributors. Overall, the patient’s renal impairment could not be fully explained by direct drug-induced nephrotoxicity alone.

## Discussion

In the present case, treatment for presumed RR-TB was initiated primarily based on a single Xpert MTB/RIF result showing rifampicin resistance with a very low bacterial load. Following the development of renal dysfunction during D2 treatment, the patient was switched to BPaLM, resulting in sequential exposure to both regimens. The causal relationship between anti-tuberculosis therapy and renal dysfunction was subsequently evaluated using the WHO–UMC causality assessment system and classified as “possible”.

This classification was supported by the temporal association between treatment initiation and onset of renal dysfunction, together with partial improvement after discontinuation of treatment and supportive management. However, interpretation remained challenging because the regimen was switched from D2 to BPaLM during the clinical course, making it difficult to distinguish the effects of drug withdrawal, new drug exposure, and the natural evolution of renal dysfunction. In addition, renal function fluctuated throughout treatment without a clear and reproducible rechallenge response, while several confounding factors-including advanced age, low BMI, frailty, and possible hemodynamic instability-may also have contributed to renal vulnerability.

Compared with the Naranjo algorithm, the WHO-UMC system was considered more appropriate in this setting because it allows broader consideration of multifactorial clinical conditions, fluctuating courses, and uncertainty regarding rechallenge and competing etiologies [5], [6], [7]. Assessment of drug-induced kidney injury in routine clinical practice remains challenging because reliable rechallenge data, validated biomarkers, and clearly established pathophysiological mechanisms are often lacking [8], [9].

Among the administered agents, none has been strongly associated with clinically significant nephrotoxicity. Current WHO guidelines, FDA labeling information, and published literature suggest that most drugs used in both D2 and BPaLM regimens have limited nephrotoxic potential. Fluoroquinolones, including levofloxacin and moxifloxacin, have been rarely associated with acute interstitial nephritis and drug-induced acute kidney injury [10], while pyrazinamide has been implicated in hypersensitivity-related renal injury in occasional reports [11]. In contrast, bedaquiline, clofazimine, cycloserine, pretomanid, and linezolid are not generally recognized as major nephrotoxic agents, with only sporadic reports of renal impairment [4], [12], [13]. Accordingly, during the D2 treatment period, levofloxacin and pyrazinamide were considered the most plausible suspected contributors, whereas the BPaLM components were considered less likely causes of direct intrinsic nephrotoxicity. Overall, the patient’s renal dysfunction could not be fully explained solely on the basis of drug-induced toxicity.

Interestingly, the most pronounced fluctuations in renal dysfunction appeared during treatment with the BPaLM regimen, during which eGFR decreased below 30 mL/min/1.73 m² and repeated hospitalizations required. However, these observations should be interpreted cautiously and does not establish causality, as renal dysfunction had already developed during D2 treatment before the switch to BPaLM. Furthermore, currently available evidence does not support clinically significant nephrotoxicity associated with pretomanid or other BPaLM components. Although approximately 53% of pretomanid metabolites are excreted in urine and clinical data in patients with renal impairment remain limited, no clinically significant pretomanid-associated nephrotoxicity has been demonstrated to date [14], [15]. Previous pharmacokinetic studies have suggested that pretomanid may increase serum creatinine through inhibition of tubular creatinine secretion without causing a true reduction in glomerular filtration rate, suggesting a pseudo-creatinine elevation rather than intrinsic kidney injury [14], [15]. BPaLM regimens are also generally considered less toxic and better tolerated than conventional multidrug-resistant tuberculosis regimens, with previous studies reporting low rates of clinically significant renal adverse events [12], [13]. Therefore, the temporal association between worsening renal function and BPaLM treatment in this case should not be interpreted as evidence of direct drug nephrotoxicity**.**

The fluctuating clinical course and partial recovery of renal function after treatment interruption, hydration, and supportive care suggest a reversible hemodynamic contribution to AKI, consistent with Kidney Disease: Improving Global Outcomes (KDIGO) concepts, and favor partially reversible renal instability over progressive intrinsic nephrotoxicity [16]. Drug-induced kidney injury is often multifactorial and may result from interactions among host susceptibility, prior renal injury, nutritional status, and cumulative exposure to multiple agents rather than toxicity attributable to a single drug alone [17], [8]. Incomplete renal recovery after the initial AKI episode during D2 therapy may also have increased susceptibility to subsequent renal fluctuations during later treatment with BPaLM. Repeated or prolonged exposure to multiple agents in patients with reduced renal reserve may further increase susceptibility to recurrent renal dysfunction [17]. Taken together, these findings suggest that the more pronounced renal impairment observed during the BPaLM treatment period likely reflected multifactorial vulnerability rather than direct nephrotoxicity attributable to the regimen itself. The D2 rechallenge also provided additional insight into the causality assessment. Although renal function had not fully recovered before reintroduction, no reproducible pattern of severe renal deterioration was observed after re-exposure, limiting the ability to strengthen a causal association between the initial D2 regimen and AKI.

Observations from this case should therefore be considered hypothesis-generating rather than confirmatory evidence of causation. From a clinical perspective, this case illustrates that worsening renal function during sequential RR-TB treatment should not automatically be attributed to the most recently introduced regimen. Careful interpretation requires consideration of the temporal sequence, previous renal injury, supportive interventions, patient-related risk factors, and the overall clinical course when making treatment decisions.

Nevertheless, this report has important limitations. Characterization of AKI was incomplete because detailed urinalysis, urine output monitoring, hemodynamic assessment, renal imaging, and advanced kidney injury biomarkers were unavailable. Other potential contributors to renal dysfunction, including dehydration severity, concomitant medications, intercurrent infections, and comorbid conditions affecting renal function, could not be comprehensively assessed [16], [17]. Consequently, non-drug-related causes of renal impairment cannot be excluded, and the pathophysiological mechanism underlying AKI in this patient remains uncertain.

This case also highlights the diagnostic uncertainty surrounding microbiological confirmation of RR-TB. The diagnosis relied on a single Xpert MTB/RIF result without confirmation by culture or repeat molecular testing. Although residual MTB DNA from prior tuberculosis treatment was considered less likely because treatment had been completed more than five years earlier, false-positive rifampicin resistance results have been reported in specimens with very low bacterial burden [18], [19]. Therefore, this report should primarily be interpreted as a case highlighting the diagnostic and therapeutic challenges encountered when renal dysfunction develops during treatment for presumed RR-TB rather than definitive evidence of nephrotoxicity during microbiologically confirmed RR-TB treatment.

In situations where the relationship between anti-tuberculosis therapy and renal dysfunction remains uncertain, individualized supportive management, optimization of hydration and nutritional status, dose adjustment according to renal function, and close laboratory monitoring remain essential to minimize toxicity while maintaining treatment safety and effectiveness. This case underscores the importance of avoiding premature attribution of renal dysfunction to a single regimen and supports individualized clinical decision-making when balancing treatment continuity against potential drug toxicity. In selected patients, if direct intrinsic nephrotoxicity from BPaLM drugs is considered unlikely after careful clinical evaluation, continuation of the shorter and less complex BPaLM regimen might still offer advantages in terms of adherence and tolerability compared with prolonged D2 therapy.

## Conclusion

This case highlights the complexity of causality assessment for renal dysfunction occurring during treatment for presumed rifampicin-resistant tuberculosis in an elderly patient. The fluctuating clinical course, sequential regimen changes**,** limited microbiological confirmation, and presence of multiple patient-related risk factors prevented the attribution of the renal impairment to a single drug or treatment regimen. Rather than relying solely on temporal associations, interpretation of renal dysfunction should integrate the overall clinical course and individual patient characteristics. Close longitudinal monitoring of renal function and individualized supportive management remain essential, particularly in older adults with limited physiological reserve.

## CRediT authorship contribution statement

**Luan Nguyen Quang Vo:** Writing – review & editing, Supervision, Resources, Funding acquisition. **Andrew James Codlin:** Writing – review & editing. **Thien Huong Nguyen:** Writing – review & editing. **Han Thi Nguyen:** Writing – review & editing. **Hoang Chinh Ha:** Writing – review & editing, Project administration, Data curation. **Thi Thanh Thuy Hoang:** Writing – review & editing, Writing – original draft, Supervision, Data curation, Conceptualization.

## Ethics declaration

Written informed consent to take part in the study and to publish the article has been obtained from all participants or their legal representatives. The privacy rights of participants have been observed.

This study was performed in compliance with relevant laws, regulatory frameworks and guidelines where the research took place. This study was conducted in accordance with CARE guidelines. Ethics committee approval was not required under relevant laws and institutional guidelines. This report describes a single, retrospectively documented clinical case and does not constitute human-subjects research. In accordance with the policy of the treating institution (Soc Trang Lung Hospital, Vietnam) and applicable national regulations, formal ethics committee review and approval are not required for an anonymized single-patient case report; all clinical care was delivered as part of routine programmatic tuberculosis management. The report was prepared in accordance with the ethical principles of the Declaration of Helsinki. Written informed consent for publication of the clinical details and accompanying images was obtained from the patient, and all potentially identifying information was removed to protect confidentiality.

This research follows the CARE guidelines and the CARE checklist.

## Ethics statement

Written informed consent was obtained from the patient for publication of this case report and accompanying clinical data. Identifying personal information has been omitted to protect patient confidentiality.

## Funding

This case report was supported by the Stop TB Partnership (STBP/TBREACH/GSA/W11-11163). The funder had no role in the clinical management of the patient, case report design, data collection, interpretation of the findings, or manuscript preparation.

## Declaration of Competing Interest

The authors declare that they have no known competing financial interests or personal relationships that could have appeared to influence the work reported in this paper.
