# Supplementary material for: Challenges in causality assessment of renal dysfunction during sequential treatment for presumed rifampicin-resistant tuberculosis in an elderly patient
Source: IDCases. 2026 Sep 9;46:e02758. doi: 10.1016/j.idcr.2026.e02758 (PMC13584142; doi:10.1016/j.idcr.2026.e02758)
Supplement: Supplementary file 1 [file mmc1.docx]

**Supplementary Table S1**. Chronological Summary of Drug Exposure, Renal Function, and Treatment Modifications

| **Variable** | **Baseline** | **Day 18** | **Day 19–21** | **Day 22** | **Day 30** | **Day 31–97** | **Day 98** | **Days 99–113** | **Day 114** | **Day 115–Month 15** |
| --- | --- | --- | --- | --- | --- | --- | --- | --- | --- | --- |
| **Body weight (kg)*** | 45 | NR | NR | NR | 45 | 45 | NR | 45 | NR | 45 |
| **Serum creatinine (µmol/L)** | 89 | 140 | NR | NR | 114 | 109–189 | 219 | 96–119 | NR | 96–124 |
| **eGFR (mL/min/1.73 m²)** | 73.2 | 42.34 | NR | NR | 54.28 | 29.46–57.3 ** | 24.65** | 51.5–66.8 | NR | 45.8–74.2 |
| **Lfx** | 750 mg QD | 750 mg QD | 750 mg QD | – | – | – |  | – | 750 mg QD | 750 mg QD |
| **Cfz** | 100 mg QD | 100 mg QD | 100 mg QD | – | – | – |  | – | 100 mg QD | 100 mg QD |
| **Lzd** | 600 mg QD | 600 mg QD | 600 mg QD | 600 mg QD | 600 mg QD | 600 mg QD | 600 mg QD | – | 600 mg QD | 600 mg QD |
| **PZA** | 1000 mg QD | 1000 mg QD | 1000 mg QD | – | – | – | – | – | 1000 mg QD | 1000 mg QD |
| **Cs** | 500 mg QD | 500 mg QD | 500 mg QD | – | – | – | – | – | 500 mg QD | 500 mg QD |
| **Bdq** | – | – | – | 400mg QD | 400mg QD | 200 mg TIW | 200 mg TIW | – | – | – |
| **Pa** | – | – | – | 200 mg QD | 200 mg QD | 200 mg QD | 200 mg QD | – | – | – |
| **Mfx** | – | – | – | 400mg QD | 400mg QD | 400mg QD | 400mg QD | – | – | – |
| **Treatment modification / supportive care** | D2 initiated | D2 continued | D2; IV fluids | Switch to BPaLM | BPaLM continued; IV fluids | Dose reduction for Bdq. Three hospital admissions | Hospitalized | All anti-TB drugs interrupted | D2 reintroduced | D2 continued |
| **Reason** | RR-TB treatment, BPaLM drugs were unavailable |  | AKI | Renal dysfunction after D2, and BPaLM drugs were available | Maintain an effective RR-TB regimen | Standard Bdq dose reduction after 2 weeks of loading dose. Hospitalized for fatigue and poor appetite. | Prolonged hospitalization due to severe renal deterioration | Severe renal deterioration | Parital renal recovery | No clear deterioration after rechallenge |
| **Other AEs** | None | None | None | None | None | None | None | None | None | None |

**Abbreviations:** QD = once daily; TIW = three times weekly. Lfx = levofloxacin; Cfz = clofazimine; Lzd = linezolid; PZA = pyrazinamide; Cs = cycloserine; Bdq = bedaquiline; Pa = pretomanid; Mfx = moxifloxacin; D2 = second DR-TB regimen; IV = intravenous; AKI = acute kidney injury; NR = not recorded/not available.

* Weight monitored monthly

** Creatinine clearance (CrCl) was estimated using the Cockcroft–Gault equation. CrCl was approximately 19.3 mL/min at a serum creatinine level of 189 µmol/L during Days 31–97 and 16.7 mL/min at a serum creatinine level of 219 µmol/L on Day 98. Based on WHO recommendations, no renal dose adjustment was required for the BPaLM drugs (bedaquiline, pretomanid, linezolid and moxifloxacin). All anti-TB drugs were discontinued from Day 99 following detection of severe renal impairment on Day 98.
